# Comparative Analysis of Transcriptional Responses to Genotoxic and Non-Genotoxic Agents in the Blood Cell Model TK6 and the Liver Model HepaRG

**DOI:** 10.3390/ijms23073420

**Published:** 2022-03-22

**Authors:** Katrin Kreuzer, Heike Sprenger, Albert Braeuning

**Affiliations:** Department Food Safety, German Federal Institute for Risk Assessment, Max-Dohrn-Str. 8–10, 10589 Berlin, Germany; katrin.kreuzer@bfr.bund.de (K.K.); heike.sprenger@bfr.bund.de (H.S.)

**Keywords:** toxicogenomics, TK6 cells, molecular markers, genotoxicity, gene expression

## Abstract

Transcript signatures are a promising approach to identify and classify genotoxic and non-genotoxic compounds and are of interest as biomarkers or for future regulatory application. Not much data, however, is yet available about the concordance of transcriptional responses in different cell types or tissues. Here, we analyzed transcriptomic responses to selected genotoxic food contaminants in the human p53-competent lymphoblastoid cell line TK6 using RNA sequencing. Responses to treatment with five genotoxins, as well as with four non-genotoxic liver toxicants, were compared with previously published gene expression data from the human liver cell model HepaRG. A significant overlap of the transcriptomic changes upon genotoxic stress was detectable in TK6 cells, whereas the comparison with the HepaRG model revealed considerable differences, which was confirmed by bioinformatic data mining for cellular upstream regulators or pathways. Taken together, the study presents a transcriptomic signature for genotoxin exposure in the human TK6 blood cell model. The data demonstrate that responses in different cell models have considerable variations. Detection of a transcriptomic genotoxin signature in blood cells indicates that gene expression analyses of blood samples might be a valuable approach to also estimate responses to toxic exposure in target organs such as the liver.

## 1. Introduction

Genotoxicity testing for regulatory purposes regularly includes several in vitro and, sometimes also, in vivo tests, e.g., aimed to analyze the micronucleus formation or the occurrence of chromosomal aberrations. The interpretation of positive findings in these assays, however, remains challenging sometimes [1,2]. As a possible alternative to the established test systems, different approaches for the use of omics technologies, especially transcriptomics, have been put forward in the past years, in order to allow for an identification of genotoxic compounds and for the stratification of genotoxic and non-genotoxic carcinogens based on their cellular effects [3,4,5,6,7]. Transcriptome analyses might therefore help to identify molecular signaling pathways that allow to distinguish genotoxins (GTX) from non-genotoxins (NGTX), which could be useful for health risk assessments [8,9]. However, omics analyses are not yet generally accepted for regulatory purposes, partly due to difficulties in standardizing experimental approaches and data processing but also related to the difficulty in defining adversity in an omics context [10,11,12].

In a previous comparative reanalysis of published literature data, we investigated microarray datasets from the metabolically competent human liver cell model HepaRG treated with the model GTX aflatoxin B1 (AB1), a mycotoxin, or the polycyclic aromatic hydrocarbon benzo[*a*]pyrene (BaP) [13]. The results highlight that, even using the same cell line and treatment, considerable differences in the obtained gene expression profiles may arise based on differences in the experimental design and data processing. The comparison of different experiments with these two GTX resulted in a rather limited overlap with respect to individual differentially expressed genes (DEGs), whereas the concordance was considerably more pronounced at the level of affected biological functions and signaling pathways [13].

In a follow-up project, we performed a comparative transcriptome analysis using mRNA sequencing in HepaRG cells with five food-relevant GTX (AB1, BaP, Las or lasiocarpine, NPYR or N-nitrosopyrrolidine, and ME or methyleugenol) and identified a 37 gene signature commonly regulated by all five compounds [14]. Further pathway analyses revealed that the obtained transcript signature was closely related to a DNA damage response and activation of the tumor suppressor TP53. Subsequent analyses with six non-genotoxic substances (3-MCPD or 3-monochloropropane-1,2-diol, ethanol, MPH or methapyrilene hydrochloride, PFOA or perfluorooctanoic acid, PFOS or perfluorooctanesulfonic acid, and WY-14643) underlined the specificity of the transcript signature for genotoxic stress. This is in line with previous works demonstrating that GTX transcript signatures or DEGs also cover pathways such as DNA damage response [5,15], cell proliferation [16], and/or TP53 signaling [17].

The liver as a major target organ of the abovementioned GTX, however, is not accessible for routine analyses in humans. Instead, blood samples can easily be obtained and analyzed for potential biomarkers of toxicity resulting from exposure to genotoxic compounds. In a previous study, Georgiadis et al. [18] demonstrated the impact of smoking on genome-wide gene expression and DNA methylation profiles in blood leukocytes of smokers, as well as the prediction of diseases linked to smoking. Therefore, in order to investigate whether the signature derived from the target organ (i.e., the liver model HepaRG) can also be used for detecting consequences of genotoxic stress in blood, we now analyzed the gene expression of lymphoblastoid TK6 cells. TK6 cells were derived from progenitor cells originally isolated from a male spherocytosis patient and are heterozygous at the thymidine kinase locus [19]. TK6 cells are a suitable and widely used model to study the TP53 signaling pathway [20,21], due to their TP53 wildtype status, to elucidate genotoxic mechanisms and modes of action [22,23,24,25] and to distinguish between genotoxic and non-genotoxic compounds [26,27,28,29,30]. Moreover, TK6 cells are a frequently used cell system for the determination of genotoxicity, as, for example, laid down for gene mutation testing in test guidelines (TGs) 476 or 490 of the Organisation for Economic Co-operation and Development [31].

Here, we present a comparative analysis of the transcriptomic effects of genotoxic and non-genotoxic food contaminants in hepatic and blood cells, highlighting similarities and differences.

## 2. Results

Lymphoblastoid TK6 cells were treated with five GTX and four NGTX compounds, and RNA sequencing was performed to obtain information about global alterations of gene expression. Bioinformatic pathway analyses were conducted to attribute effects to GTX- or NGTX-specific properties and to link biological functions and processes to the gene expression data. Furthermore, the sequencing results were compared with previously published data from the human liver cell model HepaRG, where sequencing and pathway analyses were performed using five GTX [14]. The transcriptomic responses in the two cell models were used to draw conclusions about the comparability of effects between the different in vitro models.

### 2.1. Gene Expression Analysis by RNA Sequencing in TK6 Cells

For better comparability between the data derived from the liver cell model HepaRG and the blood cell model TK6, the same concentrations were used for the treatment of the TK6 cells for RNA sequencing as previously used for the HepaRG cells, if possible. Staining with calcein-AM was performed with and without S9 mix to check for cytotoxicity (Supplementary Table S1). Except for AB1, the tests showed no cytotoxicity at the selected concentrations for the GTX substances BaP (5 μM), Las (1 μM), ME (250 μM), and NPYR (20 mM). Only AB1 had a toxic effect on TK6 cells, so that 0.05 μM was used instead of the previously used concentration in HepaRG (1 μM). For the NGTX, also no decrease in viability was found for 3-MCPD (1 mM), MPH (40 μM), PFOA (250 μM), and PFOS (50 μM).

RNA sequencing was carried out on an Illumina platform. Each treatment was sequenced in triplicate, while, for the solvent controls, five samples were analyzed and mapped to the human genome. Upon subsequent inspection of the data, samples number 15 (3-MPCD) and number 23 (NPYR) stood out with unusually reduced numbers of mapped fragments (40.4% and 32.3%, respectively). These two extreme outlier samples were therefore excluded from further analysis. After filtering for genes with low expression, 35,324 genes were retained for the subsequent analyses.

**Figure 1:**
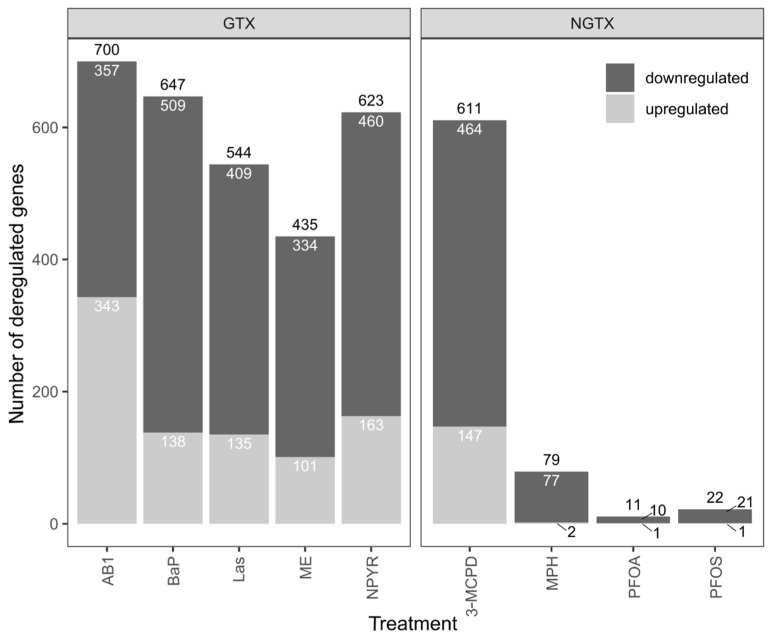
Bar plot of the number of differentially expressed genes (DEGs). Lymphoblastoid TK6 cells were treated with the genotoxins (GTX) aflatoxin B1 (AB1), benzo[*a*]pyrene (BaP), lasiocarpine (Las), methyleugenol (ME), or N-nitrosopyrrolidine (NPYR) or with non-genotoxins (NGTX) 3-monochloropropane-1,2-diol (3-MCPD), methapyrilene (MPH), perfluorooctanoic acid (PFOA), or perfluorooctanesulfonic acid (PFOS) for 24 h at high but non-cytotoxic concentrations for RNA sequencing. DEGs were analyzed by the R package DESeq2, version 1.30.1. Genes were considered deregulated with *p*_adj_ < 0.05 and a fold change |FC| > 1.5. Downregulated genes are depicted in dark gray, and upregulated genes are depicted in light gray. The genotoxic compounds yielded between 435 and 700 deregulated genes, but with the NGTX compounds, only 3-MCPD with 611 genes led to a high number of deregulated genes.

A *p*_adj_ < 0.05 and a fold change |FC| > 1.5 were chosen as the cut-off criteria for defining the DEGs. ME showed the lowest number of DEGs (435) among the GTX compounds, whereas the numbers were 544 for Las, 623 for NPYR, 647 for BaP, and 700 for AB1 (Figure 1). Among the NGTX substances, the treatment with 3-MCPD stood out with 611 DEGs, whereas MPH, PFOA, and PFOS yielded 79, 11, and 22 DEGs, respectively. It was also clearly visible that more genes were downregulated than upregulated for each treatment [14]. However, while the sequencing of HepaRG revealed clear differences in the number of DEGs with the GTX treatment, ranging from 3528 with BaP to 161 with Las, the number of DEGs in the TK6 cells was more similar when comparing the different GTX treatments.

### 2.2. Bioinformatic Analysis of Impact of Treatments on Gene Expression in TK6 Cells

**Figure 2:**
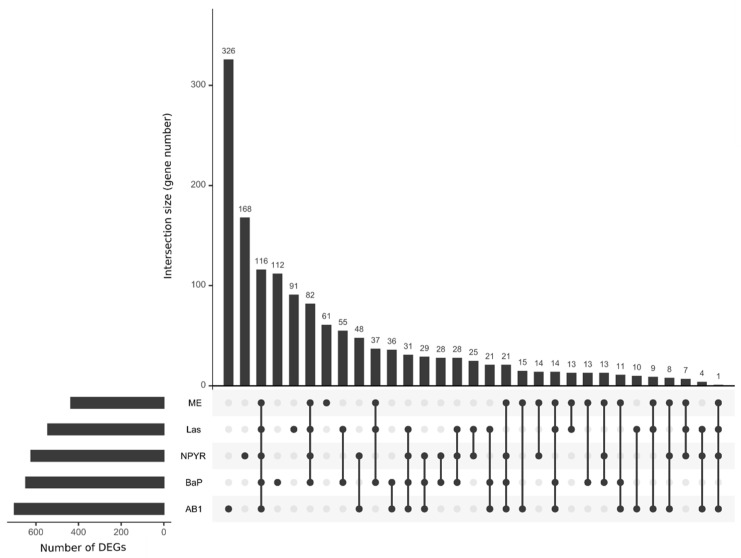
Commonalities of differentially expressed genes of GTX in an UpSet plot. RNA sequencing with lymphoblastoid TK6 cells was performed using genotoxic and non-genotoxic compounds, but only genotoxic compounds revealed many differentially expressed genes (*p*_adj_ < 0.05 and |FC| > 1.5; cp. Figure 1). These genes were visualized for overlap in an UpSet plot using R package UpSetR version 1.4.0 with the genotoxic compounds AB1, BaP, NPYR, Las, and ME. The number of DEGs is depicted on the left, the number of intersections in the upper half. The plot shows 116 genes that are commonly regulated by all five compounds.

Looking at the intersections of DEGs for treatments among the GTX compounds AB1, BaP, Las, ME, and NPYR in TK6 cells, 116 genes were significantly regulated by all five compounds (Figure 2). AB1 had the most distinct gene pattern with 326 genes differentially regulated only by AB1, followed by NPYR with 168 unique genes, BaP (112 genes), Las (91 genes), and ME (61 genes). Among the 116 commonly regulated genes, few could be linked to DNA damage and repair (e.g., BRCA2 and ANKLE1); some were connected to cell migration and adhesion (CSTA, FSCN1, ENPP2, and CD302) or to lipid or vitamin metabolism (CETP and APOD). Many protein-coding genes are related to immune responses, e.g., SERPINB10, CCR8, IGIP, LIME1, and NFKBID (Supplementary Table S2).

### 2.3. Comparison with Published Effect Marker Pattern for Genotoxicity

The purpose of the RNA sequencing experiment was to establish a transcriptional pattern for GTX or NGTX treatment in TK6 cells. In the liver cell model HepaRG, such a pattern has already been published [14]. Of the 37 published genes, 33 genes were chosen for further analysis. For the establishment of the HepaRG pattern, only significantly deregulated genes were used, which were always altered in the same direction by the different GTX test compounds.

**Figure 3:**
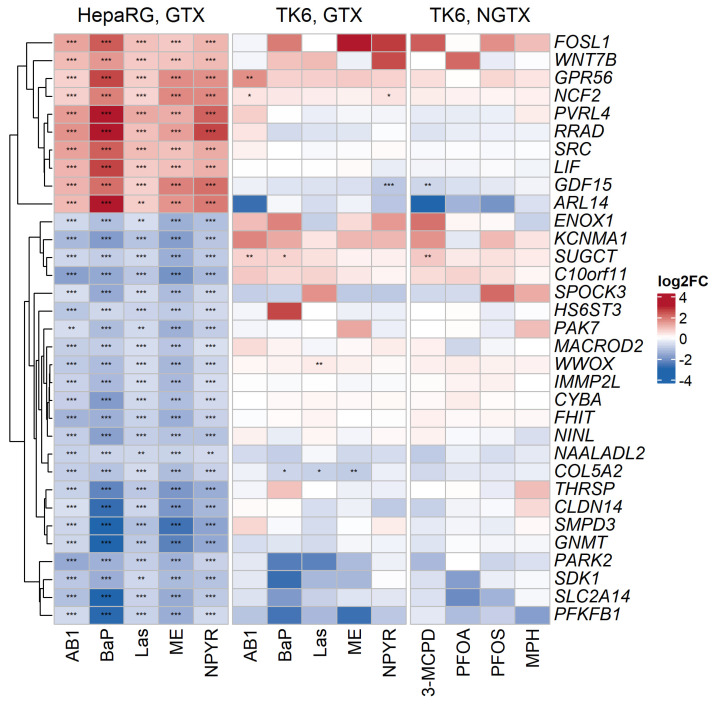
Comparison of the TK6 data with a published GTX effect marker pattern for genotoxicity. In Kreuzer et al. [14], a gene signature for GTX in the liver cell model HepaRG was published. There, 37 genes were identified, all significantly regulated in one direction with *p*_adj_ < 0.05 and |FC| > 1.5 after treatment with GTX, of which 33 genes were chosen for further analysis. The heatmap here shows a comparison of HepaRG- and TK6-derived transcriptomic data for the 33 GTX marker genes from HepaRG cells expressed as log_2_ FC values. Upregulation is depicted in red and downregulation in blue. Significance of the gene expression changes is indicated as follows: * *p*_adj_  <  0.05, ** *p*_adj_  <  0.01, and *** *p*_adj_  <  0.001.

A simple comparison of the two RNA sequencing approaches showed clear differences between HepaRG and TK6 cells in the regulation of the 33 genes of the published HepaRG GTX marker pattern. For the TK6 cells, hardly any of these 33 genes were affected by the GTX treatment; many genes were only weakly regulated (Figure 3). Only six of the genes from the HepaRG GTX pattern were also significantly altered in TK6 cells (GPR56, NCF2, GDF15, SUGCT, WWOX, and COL5A2), with three of those not corresponding to the direction of the alteration observed in HepaRG (GDF15, SUGCT, and WWOX). The expression of these genes in TK6 cells was additionally verified with independent samples by quantitative reverse transcriptase polymerase chain reaction (qRT-PCR) (Supplementary Table S3). It was also striking that there was no distinction between the GTX and NGTX effects in the mapped genes in the TK6 cells, contrasting the previously published findings with the HepaRG model [14].

### 2.4. Pathways Analysis of Gene Expression Response in TK6 Cells

**Figure 4:**
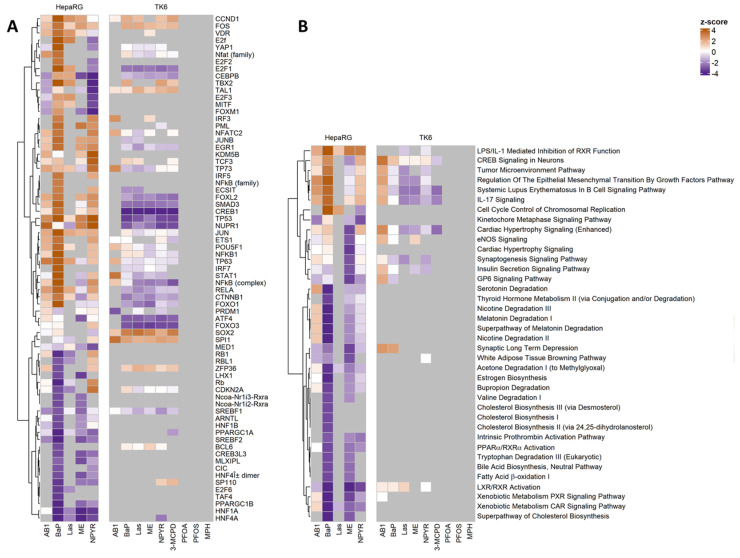
Prediction results by Ingenuity Pathway Analysis (IPA). A bioinformatic analysis with IPA indicates transcriptional factors and pathways possibly associated with the DEGs measured by RNA sequencing. In a previous study, we performed RNA sequencing in the liver cell model HepaRG with five genotoxic agents, which we have now extended to the lymphoblastoid TK6 cell model with additional non-genotoxic agents. These data from the HepaRG and TK6 models were analyzed here together using IPA software. RNA sequencing with HepaRG cells was described in Kreuzer et al. [14]. (**A**) IPA results for “upstream regulators”. The “upstream regulators” were filtered to include only transcriptional regulators. The z-score ≥ 2 or ≤−2 predicts a significantly activated or inhibited transcriptional regulator state, respectively. (**B**) IPA results for the “canonical pathways”. The z-score ≥ 2 or ≤−2 predicts a significantly activated or inhibited pathway, respectively.

Previous analyses of the transcriptomic data from GTX-treated cells showed that, despite considerable differences in the identity of individual DEGs, GTX-affected cellular functions in HepaRG cells showed a high degree of similarity across very different experiments [13]. Therefore, the pathway analysis of TK6- and HepaRG-derived gene expression data was performed using Ingenuity Pathway Analysis (IPA) to identify changes to the molecular signaling pathways and to comparatively assess the GTX effects in the different cell models (Figure 4). DEG data were used for predictions regarding “upstream regulators” and “canonical pathways”, and only |z-scores| ≥ 2 were considered to enhance the stringency. Data from the HepaRG and TK6 models were analyzed together here. A complete list of the results, including all z-scores, can be found in Supplementary Tables S4 and S5.

While, for the HepaRG cells, only GTX were examined (due to the lack of data), NGTX could also be included in the analysis for the TK6 cells. However, the IPA predictions revealed that, from the NGTX compounds, only 3-MCPD showed sufficiently high z-scores to be considered further. Strikingly, 3-MCPD was always predicted to be similar to the GTX compounds. No delineation of GTX and NGTX was therefore possible by pathway analysis. Looking at each cell line individually, the predicted “upstream regulators” and “canonical pathways” were mostly similar in direction and strength for the different treatments (Figure 4). Comparing the two cell lines showed less-predicted “upstream regulators” and “canonical pathways” in TK6 cells and, frequently, an opposite direction of the predicted direction of regulation. For example, the transcription factor CREB1 was predicted as strongly inhibited in TK6 with a z-score range from −4.227 for Las to −4.583 for NPYR, while, in HepaRG cells, CREB1 was predicted to be activated with z-scores from 0.463 (ME) to 3.373 (BaP). In the “canonical pathways” analysis, IL-17 signaling was predominantly predicted as inhibited in TK6 cells with the z-scores −1.342 (NPYR), −2.000 (ME), and −2.236 (Las and 3-MCPD), whereas the pathway was predicted to be activated in HepaRG cells with z-scores from 0.707 (ME) to 3.55 (BaP).

In particular, far less “canonical pathways” were predicted in TK6 cells, as compared to HepaRG cells. The regulated pathways predicted in TK6 cells were often connected with blood-specific features (e.g., systematic lupus erythematosus in the B-cell signaling pathway and IL-17 signaling) or with apparently irrelevant organs such as the brain (e.g., CREB signaling in neurons and the synaptogenesis signaling pathway) and heart (e.g., cardiac hypertrophy signaling). In contrast, the most striking pathways that were affected in HepaRG cells are grouped around the downregulation of lipids and cholesterol metabolism, as well as the liver X receptor (LXR).

Jointly activated transcriptional regulators in both cell lines predicted as “upstream regulators” were the two oncogenes CCND1 and FOS. The mutually inhibited regulators were CDKN2A and SREBF1, involved in the stabilization of TP53 and in sterol biosynthesis, respectively. In TK6 cells, the transcription factors FOXL2, SMAD3, CREB1, TP53, NUPR1, ATF4, and FOXO3 were very strongly predicted to be inactivated, with SMAD3, TP53, ATF4, and FOXO3 possibly associated with the stress response. Among HepaRG cells, SMAD3 and TP53 were predicted to be more strongly activated transcription factors, whereas ATF4 and FOXO3 were predicted to be only weakly involved overall.

Thus, altogether, there were more differences than similarities when comparing the two cell lines, HepaRG and TK6, considering both the “canonical pathways” and the “upstream regulators” in the pathway analysis with IPA. On the other hand, the results obtained within the individual cell lines were mostly consistent across all compounds, whether GTX or NGTX (pathway analyses in HepaRG cells refer only to GTX, as RNA sequencing data are lacking for NGTX).

### 2.5. Classification of GTX and NGTX Compounds by Transcript Markers

As detailed above, no distinction between GTX and NGTX in TK6 cells was observed regarding the expression of the 33 specific marker genes from the HepaRG model, and IPA analyses revealed also that, at the level of the pathway and regulator predictions, the TK6 cells responded substantially different to GTX treatment than HepaRG cells.

**Figure 5:**
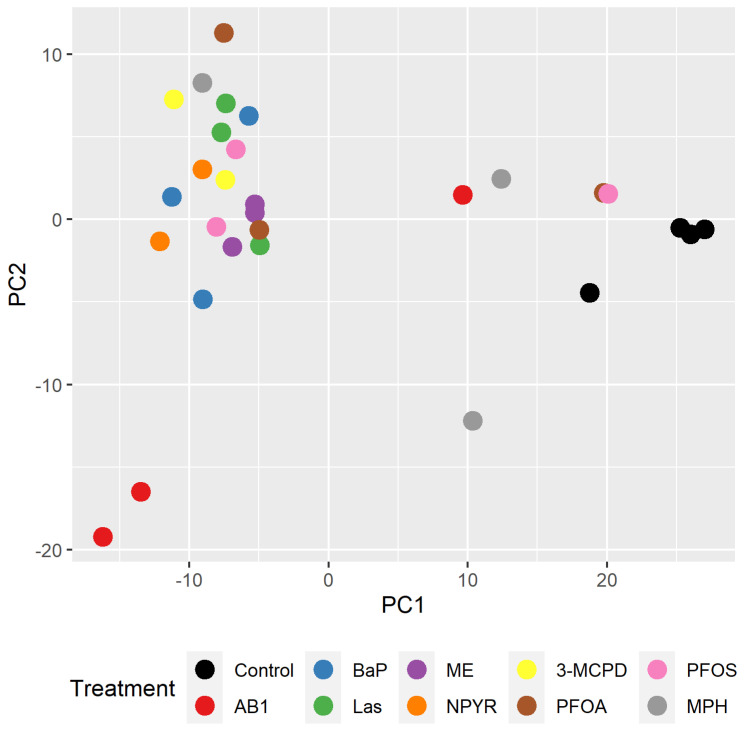
Principal Component Analysis (PCA) scores plot of transcriptome profiles for individual samples. To establish the criteria for a clear separation between GTX and NGTX compounds in TK6 cells after incubation with genotoxins AB1, BaP, Las, ME, or NPYR or with non-genotoxins 3-MCPD, MPH, PFOA, or PFOS for RNA sequencing, a PCA scores plot was created as a first attempt. PCA scores reflecting the overall structure of gene expression were performed for a subset of significant DEGs based on normalized variance-stabilized read counts. Each treatment is indicated by an individual color. Only controls cluster together, so a finer separation analysis is necessary.

Thus, a different strategy had to be chosen to establish the criteria for a clear separation between GTX and NGTX compounds in TK6 cells independent from the previous results in the liver cell model. Therefore, Principal Component Analysis (PCA) of the TK6 transcriptome data was performed as a first approach. Comparing the overall gene expression profiles in TK6 cells by PCA, only the control samples clustered together (Figure 5). Most of the other samples clustered together, with no apparent separation between treatments or differentiation between GTX and NGTX treatments. Only AB1 set apart, as well as individual samples treated with MPH, PFOA, and PFOS, which mapped more toward the control.

**Figure 6:**
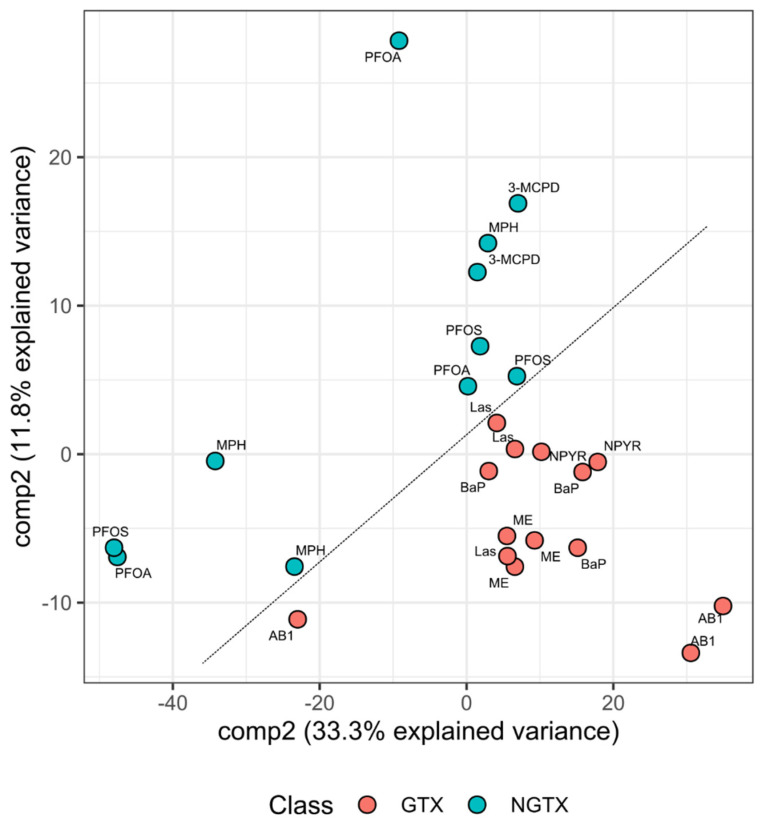
PLS-DA for separation between GTX and NGTX. To improve the separation of GTX and NGTX compounds based on TK6 RNA sequencing data with genotoxins AB1, BaP, Las, ME, or NPYR or with non-genotoxins 3-MCPD, MPH, PFOA, or PFOS, PLS-DA was conducted as an additional bioinformatics approach. For PLS-DA, R package mixOmics version 6.14.1, leave-one-out cross-validation was performed to evaluate the modeling performance, resulting in a BER of 0.35 for component 1 and averaged AUC of 0.59. A clear separation between GTX and NGTX treatments is visible and highlighted by a line. GTX compounds are depicted in red and NGTX in blue.

To improve the separation of GTX and NGTX compounds, Partial Least Squares Discriminant Analysis (PLS-DA) was conducted as an additional bioinformatics approach (Figure 6). Even though the majority of the samples tended to appear rather centered, a virtual diagonal line divided the GTX compounds into the lower right corner and NGTX compounds into the center of the graph. Leave-one-out cross-validation was performed to evaluate the modeling performance, resulting in a Balanced Error Rate (BER) of 0.35 for component 1 and an averaged Area Under the Curve (AUC) of 0.59. Thus, it appeared possible to derive a gene signature from the RNA sequencing data of the TK6 cell line.

Based on the PLS-DA to discriminate GTX from NGTX, 12 protein-coding genes were selected that were both significantly regulated by as many compounds as possible and listed as highly informative classifiers, i.e., among the top predictors of components 1 and 2 in the PLS-DA.

**Figure 7:**
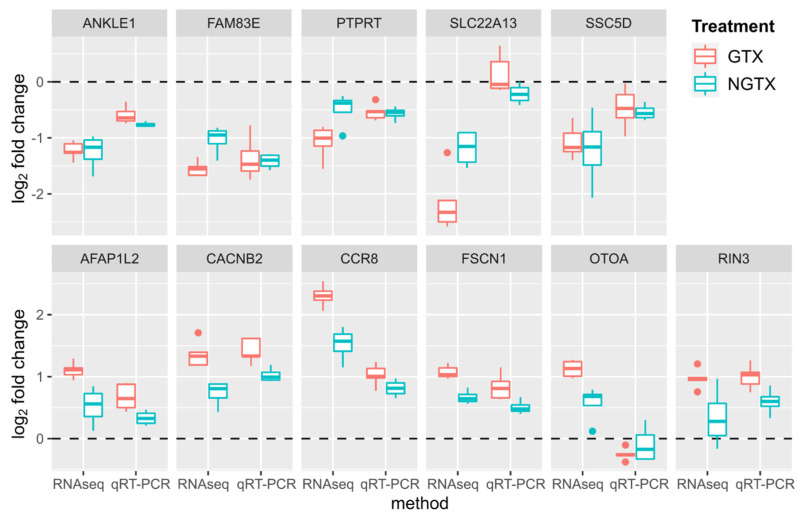
Expression changes of transcript markers in TK6 cells measured by RNA sequencing and quantitative reverse transcriptase polymerase chain reaction (qRT-PCR). Based on the PLS-DA to discriminate GTX from NGTX, 12 protein-coding genes were selected that were both significantly regulated by as many compounds as possible and listed as highly informative classifiers, i.e., among the top predictors of components 1 and 2 in the PLS-DA. Validation of the genes was performed by qRT-PCR compounds and was successful for all genes except for DNLZ. Here, the log_2_ FC values are depicted for GTX (red) and NGTX (blue), the upper panel shows downregulated genes and the lower panel upregulated genes. Overall, the qRT-PCR data were in agreement with the RNA sequencing-derived log_2_ FC values (Pearson correlation coefficient: 0.75, *p*-value: 5.24 × 10^−19^) and mostly confirmed the direction of expression.

Validation of the marker candidate genes was performed by qRT-PCR and was successful for all genes except for DNLZ. Overall, the qRT-PCR data were in agreement with the RNA sequencing-derived log_2_ FC values (Pearson correlation coefficient: 0.75, *p*-value: 5.24 × 10^−19^) and mostly confirmed the direction of expression (Figure 7; for details, see Supplementary Table S6). In particular, the gene expression patterns of AFAP1L2, CACNB2, FSCN1, and RIN3 were verified as a potential marker gene set to distinguish GTX from NGTX compounds, as these genes showed a pronounced upregulation by GTX compounds.

## 3. Discussion

The determination of the possible genotoxic potential of a chemical compound is an essential part of a toxicological evaluation. Currently, genotoxicity testing is performed using a battery of in vitro or in vivo tests [1,32,33,34,35,36]. The need for a test battery for genotoxicity arises from the limitations of individual test systems, as no single test is capable of detecting all genotoxic mechanisms. For example, the bacterial reverse mutation assay (Ames test) assesses mutagenicity, and problems with interpretation of the data may arise due to the need for an external metabolism system such as S9 mix to form reactive metabolites, with significant differences between human and rat xenobiotic metabolisms [37]. The Ames test is often complemented by tests in mammalian cells, such as the micronucleus test, which identifies chromosomal breaks and aneuploidy; here, the choice of the cell line is decisive for the outcome [2]. The progressive development in the field of in vitro cell culture, as well as in digitalization and bioinformatics, could support genotoxicity testing. Suggestions to complement the test battery used so far are in silico methods, organ-on-a-chip models, or toxicogenomics-based assays for mode of action evaluation [36,38,39,40]. It should be emphasized that classic genotoxicity assays and omics-based analyses might be used together in parallel, complementing specific shortcomings of individual approaches in order to improve the overall predictivity of genotoxicity testing.

Gene expression profiling, yielding transcript signatures, patterns, or fingerprints to distinguish genotoxic from non-genotoxic substances, has been proposed as a promising approach for almost two decades [28,41,42,43]. In particular, liver samples or in vitro liver cell models have been used for these approaches, referring to the liver as the main organ of xenobiotic metabolism. Xenobiotics are often ingested via the oral route and enter the liver through the portal vein. The liver is the main organ of xenobiotic metabolism, and a large number of phase I and phase II reactions take place there [44]. It is important to note here that many compounds, such as AB1 or BaP, must first be metabolized into an active form to have genotoxic potential [36]. In addition, blood cell models have been used in genotoxicity testing and for studying xenobiotic distribution [18,31,45]. Each model has advantages and disadvantages: animal models are expensive and lengthy, and recent approaches have tended to avoid animal testing for ethical reasons [1]. However, while in vitro models are cheap and easy to handle, they often lack adequate enzyme equipment to metabolically activate genotoxins. This can lead to contradicting results between two cell lines with different enzyme equipment or when compared with in vivo results [2,38].

Unlike the liver cell line HepaRG, lymphoblastoid TK6 cells are not equipped with high levels of xenobiotic-metabolizing enzymes [46]. In order to achieve the formation of metabolites of the original substances in the TK6 cell line, the use of exogenous metabolic systems is necessary. It is known that the different exogenous metabolic systems, such as liver microsomes, cytosol, or S9 fraction, differ in the levels of phase I and II enzymes [47]. Additionally, considering species-specific differences, such as between the most commonly used systems from rats and humans [48,49], the varying efficiencies between batches, and the cytotoxic potential of the mixes, it can be assumed that the gene expression responses will diverge, depending on the exogenous metabolizing systems used [50,51]. Godderis et al. [22] studied TK6 cells with human liver S9 mix and suggested that the S9 mix induces non-target effects on the number of DEGs, as well as on some pathways, making it difficult to identify the specific effects. Buick et al. [26] examined the transcript response of TK6 cells to increasing concentrations of GTX and NGTX compounds, including AB1 and BaP, in the presence of rat liver S9, addressing also the influence of S9 to the postulated gene signature TGx-28.65. Comparisons between controls with and without S9 showed no difference in relative survival, and classification based on signature was correct. It is concluded that the use of S9 results in changes in gene expression, but these changes do not appear to affect the ability to recognize a non-genotoxic signature or cause an independent genotoxic classification [26,52].

In Kreuzer et al. [14], we previously published a gene signature to discriminate GTX from NGTX in the human liver cell model HepaRG, which is metabolically competent and similar to human hepatocytes in its enzyme expression [53]. The gene signature, distinct from previous approaches, was determined by the overlap of the differentially expressed genes of five food-relevant genotoxins. The 37 genes of the pattern were significantly regulated by all treatments with high but non-cytotoxic concentrations, the direction of regulation was always the same for each gene, and the specificity was demonstrated using various non-genotoxic agents [14]. However, liver samples from humans are difficult to obtain, and genetic signatures from non-accessible target organs are of very limited use for biomonitoring approaches in humans. Therefore, to analyze GTX-induced gene expression changes in an easily accessible tissue, and to address the question of transferability of the obtained liver gene expression signature to another cell model, we selected the TP53-competent lymphoblastoid cell model TK6 as the representative model for blood cells. The protein TP53 plays a crucial role in the regulation of DNA damage and repair to prevent mutations, but the TP53 gene (encoding TP53) is known to be mutated and/or silenced in many immortalized cell lines [54,55].

Comparative gene expression analyses of different in vitro models show a dependence of the results on the individual cell models, often due to differences in metabolic processes. Hart et al. [56] compared the gene expression of 115 genes involved in xenobiotic metabolism in HepaRG and HepG2 human hepatoma cells with human primary hepatocytes and liver tissues and showed that the gene expression of HepaRG cells is more similar to human primary hepatocytes and human liver tissue than to HepG2 cells. Since HepG2 cells have little metabolic activity, HepaRG cells were also considered by Jennen et al. [57] and Berger et al. [58] to be the more suited liver cell model for exposure and metabolism studies.

Despite the described differences in different liver cell models, a similar response to xenobiotic substances seems to be present in cell models of different species. The impact of environmental pollutants in the liver hepatocellular carcinoma cell line HepG2 and the human leukemia cell line HL-60 cells was investigated by Song et al. [45], suggesting that transcriptomic responses are toxicant-specific, indicating a characteristic molecular signature for each pollutant group. Specific studies of the responses between cell models have not been performed, but with a gene signature of 265 genes, there appeared to be many similarities between HepG2 and HL-60. Using machine learning, Li et al. [59] identified the TGx-DDI biomarker consisting of 64 genes, which responded specifically to DNA damage. The development of this biomarker started with transcriptomic profiles for TK6 cells exposed to 28 model chemicals representing a wide range of well-characterized modes of action, followed by validation studies with further chemicals and including several methods for gene expression analysis. The compatibility of the TGx-DDI biomarker set with another cell line was tested successfully using HepaRG cells [60]. HepaRG samples were analyzed using an Ampliseq whole-transcriptome profiling approach and aligned with the TK6-derived TGx-DDI biomarker set, resulting in accurate predictions.

Even though we performed treatment and gene expression analyses as similar as possible in HepaRG and TK6 cells, most of the measured 33 genes of the HepaRG pattern were not significantly expressed in the TK6 cells. Therefore, it is particularly important to look not only at the individually regulated genes but also especially at the overall regulated signaling pathways for a statement about the comparability between the liver cell model HepaRG and the blood cell model TK6. New insights into signaling pathways and/or the differences and similarities in various in vitro models could contribute to improving the mode of action and adverse outcome pathways (AOPs). AOPs are a relatively new approach that could be used for risk characterization of genotoxicity [61].

In this study, a pathway analysis was performed using IPA on “upstream regulators” and “canonical pathways”. Here, the DEGs of both RNA sequencing analyses with HepaRG and TK6 cells were uploaded for the prediction of transcription factors and classical pathways based on the given genes. The results showed that the predictions for each cell line were consistent across the different treatments, but when comparing the two cell lines, the direction of prediction was mostly the opposite. The most surprising result here was the prediction of the transcription factor TP53. Additionally known as the “guardian of the genome”, this tumor suppressor accumulates in the cell after DNA damage and consequently regulates, amongst other functions, gene expression for DNA repair and apoptosis [62]. The addition of GTX to a cell system would be expected to increase the TP53 protein levels, and IPA analysis could conceivably predict activation of the TP53 transcription factor. This is the case for HepaRG cells, whereas, for TK6 cells, IPA predicts inhibition of the TP53 protein. It has been shown that *TP53* is not mutated and that the TP53 signaling pathway is wildtype and activated by the incubation with GTX in HepaRG [4,63,64] and TK6 cells [28,65]. Therefore, an actual inhibition of TP53 protein seems unlikely. Classical genes related to the TP53 pathway, such as ATF3, CDKN1A, or GADD45A, are described as robustly upregulated in TK6 cells upon the application of stress agents [21,23,26,66]. In our study, GADD45A was significantly downregulated by NPYR, while ATF3 and CDKN1A were downregulated by BaP, Las, NPYR, and 3-MCPD, respectively. Shortly after, e.g., DNA damage, TP53 undergoes protein modifications, the half-life of the TP53 protein and its concentration in the cell are increased, and enhanced binding of TP53 to the target gene promoters occurs [62]. While, in our study, the incubation time was 4 h plus a 20 h recovery period due to the use of S9 mix, the incubation time often has been either 4 h [26,66] or 24 h [21,23] in previous studies. The differences in the TP53 pathway response might have occurred due to the recovery phase. While the other studies recorded the effects of genotoxic stress during continuous exposure to GTX, in our work, the impact of GTX and its metabolites after 4 h of exposure and 20 h of action was measured. With the time point chosen, the phase of TP53 activation could have already ended, which is why the genes ATF3, CDKN1A, and GADD45A were downregulated, and TP53 was predicted by IPA to be inhibited. In HepaRG cells, where the incubation time was 24 h, activation of the TP53 signaling pathway was predicted, leading to cellular effects such as DNA repair programs [14]. While some people might argue that the recovery phase could be perceived as a drawback of our experimental setup for comparing the results with previous studies, it should be emphasized that the data gained in the present study may be more realistic with respect to future applications of marker gene expression analyses from human blood samples, where a time point of sampling distant from the time point of exposure appears likely. Nonetheless, much more transcriptomic data on the response to GTX and NGTX in in vitro systems is required to reach a future routine application of transcriptomic approaches for genotoxicity testing. In addition to analyses of comparability with classic genotoxicity assays, this also includes the testing of further compounds in additional cell lines at a wide range of concentrations and time points.

Within the present study, we were able to distinguish between GTX and NGTX compounds in TK6 cells based on a bioinformatic analysis of the transcriptional data. The possibility to discriminate between GTX and NGTX substances and/or carcinogens based on an mRNA transcript signature has been demonstrated in different cell types and tissues. Within that context, liver cell models such as HepaRG [17,67] and HepG2 [6,68], as well as comparative approaches with different cell lines [4,57,69], have been used frequently. Moreover, mouse liver [5,70] and rat liver [71,72] have been analyzed. In addition, blood cell models have been used [42,45], including TK6 cells [22,26,30].

In our approach, using the PLS-DA approach, we successfully determined 11 genes that can be used for a gene signature to discriminate GTX from NGTX in TK6 cells. Only a few of those have been previously listed in the literature as potential marker genes, primarily for cancer. The expression level of AFAP1L2, involved in signal transduction, has been described as a potential tumor marker in soft tissue tumors [73], while endonuclease-encoding ANKLE1, which plays a role in DNA damage and repair, has been suggested as a breast cancer marker [74]. FSCN1 has been proposed for renal cells, as well as for tongue squamous cell carcinoma; this gene encodes a member of the fascin family of actin-binding proteins [75,76]. However, none of the 11 genes are described in the biomarker set TGx-DDI [59], nor are they contained in other published signatures obtained from GTX-treated TK6 cells [23,29,30,66,77,78]. With the exception of ANKLE1, the classifiers are not linked to DNA damage and repair but express proteins related to, for example, voltage-gated calcium channels (CACNB2), the inner ear (OTOA), or urate uptake in the kidney (SLC22A13). This is different from the HepaRG signature, where within the elaborated 37 genes, many were associated with the DNA damage response and/or cell death and others with cell cycle progression or cell adhesion [14].

Thus, these contrasts highlight the differences between the two cell lines considered and suggests that a uniform gene signature in vitro for genotoxicity is difficult to achieve and the selected marker genes require detailed screening when applied to another cell system.

## 4. Materials and Methods

### 4.1. Chemicals

AB1 was obtained from Sigma-Aldrich (Steinheim, Germany; catalog no. A6636; purity ≥ 98%; lot 028M4088V), BaP from Supelco (Bellefonte, PA, USA; catalog no. 48564; analytical grade, lot LC15023V), Las from PhytoLab (Vestenbergsgreuth, Germany; catalog no. 80412; analytical grade; lot 12080), ME from Sigma-Aldrich (catalog no. 04607; analytical grade; lot BCBV6695), and NPYR also from Sigma-Aldrich (catalog no. 158240; purity ≥ 99%; lot MKCB3427).

3-MCPD was purchased from Sigma-Aldrich (catalog no. 10721, purity 98%, lot MKCC7598), MPH was obtained from Supelco (catalog no. 44-2641; purity 99.9%, lot LC1217V), PFOA from Sigma-Aldrich (catalog no. 171468; purity 96%; lot 11419LE), and PFOS from abcr (Karlsruhe, Germany; catalog no. AB128838; purity 97%; lot 1307952).

For use in cell viability assays, 100× stock solutions of the test chemicals were prepared in dimethyl sulfoxide (DMSO; AppliChem, Darmstadt, Germany). DMSO concentrations in the experiments therefore never exceeded 1%. 3-MCPD was diluted in culture medium. For use in the RNA sequencing experiment, 1000× stock solutions were prepared in DMSO, while 3-MCPD was diluted in the medium and NPYR was used in its undiluted form.

### 4.2. Cell Culture

The human lymphoblastoid cell line TK6 (CLS, Eppelheim, Germany) was cultured in RPMI 1640 Medium with L-glutamine and 2.0 g/L NaHCO_3_ (PAN-Biotech, Aidenbach, Germany) supplemented with 10% fetal bovine serum (Biochrom, Berlin, Germany), 100 U/mL penicillin, and 100 μg/mL streptomycin (Capricorn Scientific, Ebsdorfergrund, Germany). The cells were maintained in suspension culture at a density between 0.5 × 10^5^ and 1 × 10^6^ cells/mL. For all studies, 1 × 10^5^ cells/mL were seeded and cultured for 48 h prior to treatment at 37 °C and 5% CO_2_. For incubation with the test compounds, the cells were counted with a hemocytometer EVE (NanoEnTek, Seoul, Korea) using trypan blue (NanoEnTek) and diluted to 0.5 × 10^6^ viable cells/mL with fresh medium.

TK6 cells were treated with genotoxic and non-genotoxic compounds in the presence of 1% induced rat liver S9 mix from male Wistar rats for metabolic activation. Rat S9 mix was prepared as previously described [49]. Animal experiments for rat S9 preparation were performed in accordance with the European laws and with the consent of the Regional Office for Health and Social Affairs Berlin (LaGeSo), approval numbers H0256/02 (treatment of rats) and T0258/02 (killing of animals and isolation of organs). The activation solution consisted of 33 mM potassium chloride (Merck, Darmstadt, Germany), 8 mM magnesium chloride (Merck), 4 mM NADP (Carl Roth, Karlsruhe, Germany), 5 mM glucose-6-phosphate (Carl Roth), and 10% rat S9 fraction diluted in 15 mM sodium phosphate buffer (pH 7.4) (Merck) and was preincubated with test compounds before adding TK6 cells for treatment as follows: According to the calculated final volume in the experiment, the activation solution and cell culture medium containing the appropriate concentration of the test compound (max. 1% of test compound stock solution in DMSO dissolved in the medium) were mixed at a 1:1 ratio and incubated at 37 °C. After 30 min, cell suspension was added to the result in a final concentration of 0.4 × 10^6^ cells/mL (i.e., 20% preincubation mix and 80% of a 0.5 × 10^6^ cells/mL cell suspension), and incubation started for 4 h, followed by a recovery phase of 20 h. For recovery, cells were centrifuged at 400× *g* for 5 min, the supernatant was removed carefully, and cells were washed three times carefully with prewarmed (37 °C) phosphate-buffered saline (PBS) and a centrifugation step at 400× *g* for 5 min. Subsequently, fresh culture medium was added, and the cells were put in the incubator at 37 °C until a total incubation time of 24 h.

### 4.3. Cell Viability Assay

Cell viability was measured using a live staining with the fluorescent marker calcein-AM (PromoCell, Heidelberg, Germany). Three controls were included: a medium control with culture medium instead of treatment, a solvent control with 1% DMSO, and a positive control with heat-inactivated cells (95 °C, 20 min). Following treatment and recovery, with and without exogenous metabolic activation, 1.5 μL calcein-AM (10 μM in DMSO) was applied and incubated for 1 h at room temperature in the dark. Cells were analyzed using a flow cytometer (BD Accuri C6, Becton Dickinson, Franklin Lakes, NJ, USA). A total of three biological replicates were measured and evaluated by calculating the mean of the live staining calcein-AM. Only non-cytotoxic concentrations with a viability of at least 80% compared to the control were used for gene expression analyses.

### 4.4. RNA Isolation

Cells were washed twice with ice-cold PBS and harvested with RLT buffer containing β-mercaptoethanol (Qiagen, Hilden, Germany). RNA isolation was performed with the RNeasy Mini Kit (Qiagen). For RNA quality and quantity determination, an Agilent 2100 Bioanalyzer with RNA Nano Chips (Agilent Technologies, Santa Clara, CA, USA) was used. RNA isolation and characterization were conducted according to the manufacturers’ protocols.

### 4.5. RNA Sequencing

Total RNA sequencing was performed at CeGaT (Tübingen, Germany). In brief, libraries were prepared from 100 ng of RNA using the TruSeq Stranded Total RNA kit (Illumina, San Diego, CA, USA). Sequencing was performed on a NovaSeq 6000 system, 2 × 100 bp (Illumina). Demultiplexing of sequencing reads was accomplished using Illumina bcl2fastq (version 2.20). Depths of ~60–140 million reads were generated for each sample (see Supplementary Table S7 for details). The raw RNA sequencing data are available from GEO under accession number GSE185362. Adapters were trimmed by Skewer version 0.2.2 (Jiang et al. 2014), and the data quality was assessed by FastQC version 0.11.5 [79]. Reads were aligned to the human genome (hg19) and counted per gene ID using STAR (version 2.7.3) [80].

### 4.6. Quantitative Reverse Transcriptase Polymerase Chain Reaction (qRT-PCR)

qRT-PCR was used to investigate the transferability of a published gene signature for genotoxicity from HepaRG cells to TK6 cells (of the 37 published genes, 33 genes were chosen for further analysis), as well as to verify the TK6 RNA sequencing data. Cultivation, treatment and RNA isolation were performed as described above. The transcription of RNA into cDNA was conducted with the High-Capacity cDNA Reverse Transcription Kit (Applied Biosystems, Foster City, CA, USA) according to the manufacturer’s protocol.

qRT-PCR was carried out with the Maxima SYBR Green/ROX qPCR Master Mix (Thermo Fisher Scientific, Waltham, MA, USA) on a ABI7900HT (Thermo Fisher Scientific). The thermal cycling procedure started with an initial denaturation at 95 °C for 15 min. This was followed by 40 cycles of denaturation for 15 s at 95 °C and primer binding and elongation for 1 min at 60 °C. The procedure ended with a final elongation at 60 °C for 15 min and the addition of a dissociation curve step. Primers were purchased from Eurofins Genomics (Ebersberg, Germany); the sequences are shown in Supplementary Table S8. ACTB, GAPDH, and GUSB were used as housekeepers and geometrically averaged.

### 4.7. Bioinformatic Analysis and Statistics

After removing samples with poor RNA sequencing data quality (samples 15 and 23) and genes with low expression (sum of reads across all samples below two), the retained genes were analyzed by the R package [81] DESeq2 version 1.30.1 [82] using the default settings for estimation of the size factors and dispersion. Negative Binomial GLM fitting and Wald statistics were applied to test for differential gene expression between each treatment and control condition, respectively. The false discovery rate (FDR) was used to control for multiple testing [83]. Only genes with a *p*_adj_ < 0.05 and a |FC| > 1.5 were identified as DEGs and included in further analyses. Variance stabilizing transformation was applied prior to probabilistic Principal Component Analysis (ppca) on pareto-scaled and centered data by the R package pcaMethods version 1.82.0 [84]. Heatmaps were generated by the R package ComplexHeatmap version 2.9.3 [85] using the default settings. Intersections of gene sets were visualized as UpSet plots using R package UpSetR version 1.4.0 [86]. 

PLS-DA was performed to discriminate GTX and NGTX compounds using R package mixOmics version 6.14.1 [87] and select marker candidates for qRT-PCR verification. The performance of the PLS-DA model was evaluated by leave-one-out cross-validation and measured by the BER and AUC using the maximum distance.

### 4.8. Pathway Analysis

For the pathway analysis, significant DEG results were evaluated by using the software IPA (version 65367011; QIAGEN, Redwood City, CA, USA; www.qiagenbioinformatics.com/products/ingenuity-pathway-analysis, accessed on 17 March 2022). We performed the IPA Canonical Pathway and Upstream Regulator Analysis to investigate potentially effected pathways and transcriptional regulators. Fisher’s exact test was used to estimate the statistical significance of the predictions, and significance was assumed at *p*-value < 0.05. Upstream regulator results were filtered to include only transcriptional regulators. A z-score ≥ 2 or ≤−2 predicted a significantly activated or inhibited transcriptional regulator state, respectively.

## 5. Conclusions

Overall, our study, including a comparative RNA sequencing approach of GTX-treated human liver and GTX- and NGTX-treated blood cells, presents a transcriptomic signature for genotoxin exposure in a human TK6 blood cell model. The data showed that responses vary widely in different cell models. Nevertheless, it was possible to detect a transcriptomic genotoxin signature in the blood cell model. This suggests that gene expression analyses of blood samples could be a valuable approach to also evaluate responses to toxic exposure in target organs, such as the liver. This way, the determination of transcript signatures from human blood cells might be of future use for risk assessment, possibly to complement genotoxicity testing, as transcriptional alterations in blood cells could be used for indirectly monitoring genotoxic stress in target organs not accessible for such analyses.

## Data Availability

The RNA sequencing data are available from GEO under accession number GSE185362.

## Supplementary Materials

The following supporting information can be downloaded at https://www.mdpi.com/article/10.3390/ijms23073420/s1.
